# The social defeat hypothesis of schizophrenia is more topical than ever. Reply to Schalbroeck

**DOI:** 10.1017/S0033291720005176

**Published:** 2023-01

**Authors:** Jean-Paul Selten

**Affiliations:** 1 School for Mental Health and Neuroscience, University of Maastricht, Maastricht, The Netherlands; 2 Rivierduinen Institute for Mental Health Care, Leiden, The Netherlands

We thank Mr. Schalbroeck for his interesting and well-written letter in which he raises two points: (i) the authors of the social defeat hypothesis defined the concept of defeat inconsistently; (ii) since social defeat is difficult to measure, the hypothesis is of limited scientific value (Schalbroeck, 2020).

Firstly, we prefer the original definition of social defeat: the long-term experience of a subordinate position or outsider status (Selten & Cantor-Graae, 2005). In 2013 we defined social defeat as ‘the negative experience of being excluded from the majority group’ to make clear that we refer to an unwanted position, not to the outsider status of individuals such as expats or avant-garde artists (Selten, van der Ven, Rutten, & Cantor-Graae, 2013). However, we think that the experience of a subordinate position, with the accompanying lack of autonomy, is important too.

Secondly, with reference to the measurement of social defeat, we would like to distinguish between the measurement of (i) outcomes that often indicate the experience of social defeat (such as a low level of education, single status, unemployment, low income and poor housing) and (ii) the experience of social defeat itself. While the measurement of these outcomes is not particularly difficult, that of the experience of social defeat itself constitutes a problem. One can ask persons to evaluate their status, but some persons will not admit to others that they are unsuccessful. They may even not admit it to themselves. Scales to measure self-esteem, for example, do not indicate lower global self-esteem among ethnic minority youth in western countries (Verkuyten, 1994).

The social defeat hypothesis is an interpretation of a pattern of epidemiological findings. Researchers who question the value of a hypothesis based on an interpretation may be misled by the dominance of neuroscience and its emphasis on measurement. Interpretation is vital to a discipline like psychiatry, which combines neuroscience with social science.

According to the most influential philosopher of science, the utility of a hypothesis is not determined by measurability, but by falsifiability (Popper, 1963). We developed falsifiable hypotheses (e.g. increased psychosis risk for individuals with a non-heterosexual orientation or for those with an autism spectrum disorder; increased amphetamine-induced dopamine release in the striatum of persons with hearing impairment), which survived the pertinent tests and yielded valuable information to the field (e.g. Gevonden et al., 2014a; Gevonden et al., 2014b; Selten, Lundberg, Rai, & Magnusson, 2015). On the basis of our theory, one can develop more falsifiable hypotheses, such as increased psychosis risks for Aboriginal populations in Australia, New Zealand and Canada, and for individuals with a gender identity disorder. One could criticize the hypothesis for not enabling us to predict the outcome for an individual, but such hypotheses are scarce in psychiatry.

Mr. Schalbroeck questions the possibility to establish whether a group is differentially exposed and provides the example of autism, but most persons with this disorder do feel the need for social participation. As for groups of people with black skin colour, low IQ, hearing impairment, autism or a non-heterosexual orientation, their diminished access to mainstream society is beyond any reasonable doubt. One could argue that this method entails the risk of an ecological fallacy, which would be the case if, for instance, successful migrants were found to be at an equal risk as non-successful migrants, but the available evidence does not support this interpretation. Morgan et al. (2008) demonstrated for African-Caribbeans a clear linear relationship between the number of outcomes that reflect social defeat and the risk of psychosis.

In 2005 we were unaware of a theory developed by the epidemiologist Marmot, who proposed that persons lower in the hierarchy are more likely to be affected by a wide range of diseases, a phenomenon he coined status syndrome (Marmot, 2005). He argued that key factors related to a person's position in the hierarchy include a subjective sense of control over one's life (autonomy) and the opportunity for social participation. The resemblance between the social defeat hypothesis and Marmot's status syndrome is striking. Both theories are based on the idea that man is a social animal that does not endure subjugation or social exclusion. According to Marmot low status leads to increased activity of the sympatho-adreno-medullary axis and the hypothalamic-pituitary-adrenal axis (HPA-axis), which leads in turn to metabolic changes and reduced immunity. While his theory does not pretend to explain why a person develops a particular disorder, the social defeat hypothesis posits that dopamine dysregulation in response to social defeat may lead to schizophrenia. Given the influence of the HPA-axis on dopaminergic pathways, one could hypothesize that increased sensitivity of these pathways (in particular the nigrostriatal pathway) to stimulation by glucocorticoids may contribute to the vulnerability to schizophrenia.

In order to avoid any misunderstanding, we would like to point out that a ‘parsimonious explanation for several psychosis risk factors’ is not equivalent to ‘the experience of social defeat explains everything’. As we already observed in 2005, social defeat is not always followed by the development of a psychiatric disorder and is also a risk factor for depression and addiction. Other factors, including genetic vulnerability, would determine the nature of the outcome (Selten & Cantor-Graae, 2005).

Finally, since defeating experiences have probably more impact in societies with a higher level of socio-economic inequality, the social defeat hypothesis is more topical than ever.
